# A Severe Disc Herniation Mimics Spinal Tumor

**DOI:** 10.7759/cureus.36545

**Published:** 2023-03-22

**Authors:** Eric Chun-Pu Chu, Andy Lin, Kevin Hsu Kai Huang, Gordon Cheung, Wai Ting Lee

**Affiliations:** 1 Chiropractic and Physiotherapy Department, New York Medical Group, Hong Kong, HKG

**Keywords:** chirpractor, chiropractic, chiropractic therapy, disc prolapse, lumbar disc herniation

## Abstract

Lumbar disc herniation (LDH) is prevalent among adults aged 25-55 years who spend a considerable proportion of their time sitting or standing with heavy workloads. We report the case of a 33-year-old male waiter with severe LDH, causing compression of the nerve roots and spinal cord with neurological dysfunction, who presented to a chiropractic clinic. Magnetic resonance imaging (MRI) revealed a radiological differential diagnosis comprising LDH and an epidural mass lesion. To rule out serious pathology, a second MRI with contrast was ordered, which confirmed the diagnosis of severe LDH. Diagnosing large LDH may be challenging, and severe disc herniation often mimics spinal tumors. This study offers insights into the differential diagnosis of LDH and spinal tumors, as well as the design of a treatment strategy for severe LDH in the chiropractic clinic.

## Introduction

The incidence of sciatica is between 13% and 40%, and the most prevalent cause of sciatica in adults is lumbar disc herniation (LDH) [1]. LDH is prevalent among adults aged 25 to 55 years who spend a considerable proportion of their time sitting or standing with heavy workloads [2]. LDH is an orthopedic condition characterized by the displacement of an intervertebral disc from its anatomical position, usually caused by trauma or degenerative changes [2]. This displacement can occur as a result of a traumatic injury or degenerative changes due to the aging process [3]. This displacement can cause pain and disability in the spine and may require medical treatment [3]. Symptoms of lumbar disc herniation include back pain, numbness, or weakness in the area of the body to which the nerve travels, and possible loss of feeling in the genital/rectal region [4]. Treatment may include chiropractic, physical therapy, medications, epidural steroid injections, and sometimes surgery [4].

The clinical presentation is challenging to distinguish from other causes of lumbar canal stenosis, including disc herniations, synovial cysts, epidural hematomas, and tumors. It is clinically known to be a chronic condition with a high rate of relapse and limited potential for successful outcomes from radical treatments, leading to long-term patient suffering [5]. Consequently, medical professionals face an ongoing challenge in developing effective treatments and rehabilitation techniques to reduce the occurrence of recurrent LDH attacks [5]. We report the case of a 33-year-old male waiter with severe LDH, causing compression of the nerve roots and spinal cord with neurological dysfunction, who presented to a chiropractic clinic. This study offers insight into the differential diagnosis of LDH and spinal tumors in a chiropractic clinic, as well as the design of a treatment strategy for severe LDH.

## Case presentation

A 33-year-old male waiter has been experiencing low back pain for the past month, mostly on his left side. He typically stands for 10 hours at work, and the pain has a pulling sensation that radiates from the left gluteal region, lateral hip, and calf. The onset of pain was insidious and aggravated by prolonged sitting for more than 30 min. Furthermore, he experienced a pulling sensation in his left hip flexor when climbing the stairs. He denied any change in bladder function, sensation in the saddle area, or sexual sensation. The patient had tried traditional Chinese medicine and acupuncture one month ago; however, the pain only worsened as he experienced numbness in his left lateral calf. He was unable to work for over one month. He visited the emergency department one week prior, and the radiology department reported negative findings. He was diagnosed with lumbar sprain/strain where he received a corticosteroid injection and a prescription for hydrocodone-acetaminophen every four to six hours. The patient was subsequently referred to an orthopedic surgeon for surgical treatment. The patient then sought chiropractic therapy for a second opinion of treatment.

The patient was a nonsmoker with a history of low back pain that began eight years ago when he was performing 10 kg deadlifts. He went to the emergency room and was prescribed codeine, after which his symptoms disappeared. He is currently taking serotonin and benzodiazepines for insomnia. In the past, he has had no other health complaints, and no record of any other injury, surgery, or trauma.

**Figure 1:**
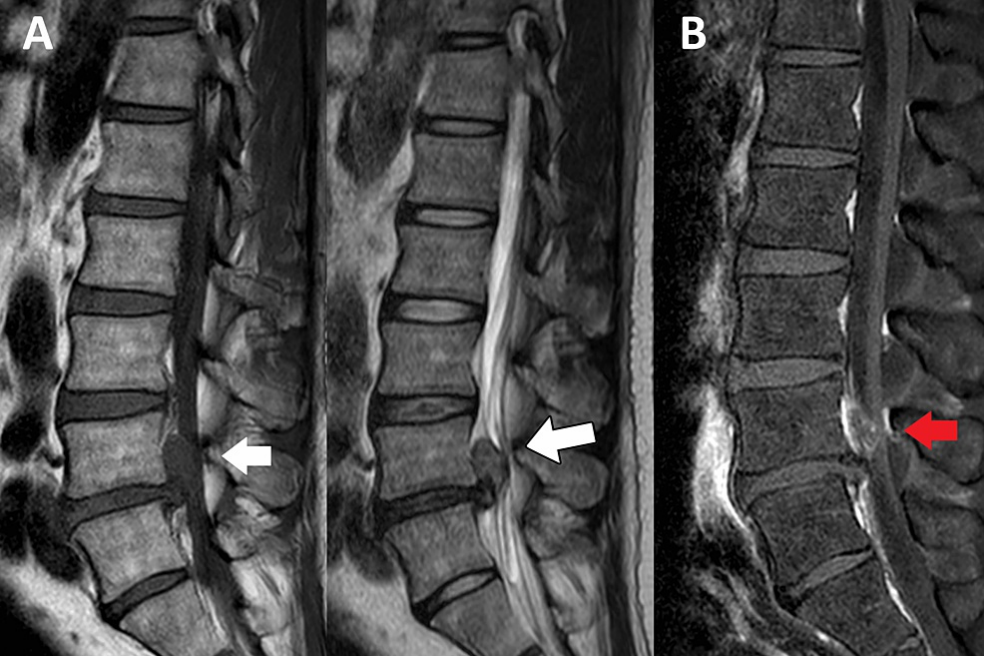
Magnetic resonance imaging of lumbar spine A) Magnetic resonance imaging of lumbar spine without contrast identifies a symmetric epidural mass lesion measuring 1.33 x 1.00 x 1.63cm at the left superior aspect of the L4/L5 disc. It shows T1W hypointense and T2W hyperintense signal (white arrow) and slight signal difference with the original disc. No bone erosion or destruction and the mass lesion compresses the spinal nerve cord. The differential diagnosis includes lumbar disc herniation and spinal tumor. B) Magnetic resonance imaging of lumbar spine with gadolinium contrast identifies that the L4/5 epidural mass lesion shows no contrast enhancement (red arrow) since disc is avascular. This implies the mass lesion is likely to be a superior migration of L4/5 disc herniation.

The patient presented with antalgic gait at the chiropractic clinic. Upon examination, orthopedic examinations revealed aggravated pain with lumbar flexion and extension in the passive range of motion. Additionally, the slump test was positive on the left side, and the Kemp test was positive for both the right and left hip. The left straight leg raise was positive at 45°. Muscular palpation revealed tenderness in the left quadratus lumborum, gluteal muscles, and hip flexors. Furthermore, reduced sensation was identified in the left L5 dermatome, and left L4 (knee flexors) strength was graded 4 on a scale of 0-5. Osseous palpation revealed joint restriction at the L4/5 and sacroiliac joints. The differential diagnoses were lumbar disc herniation, synovial cysts, and epidural hematomas. To confirm the diagnosis, a lumbar MRI (Figure 1A) was ordered and the results revealed an epidural mass lesion measuring 1.33 x 1.00 x 1.63 cm at the left superior aspect of the protruded disc. To rule out a mass lesion, the patient underwent a contrast MRI scan (Figure 1B) in the clinic. After further evaluation, the results confirmed superior migration of the lumbar disc herniation at the L4/5 level.

**Figure 2:**
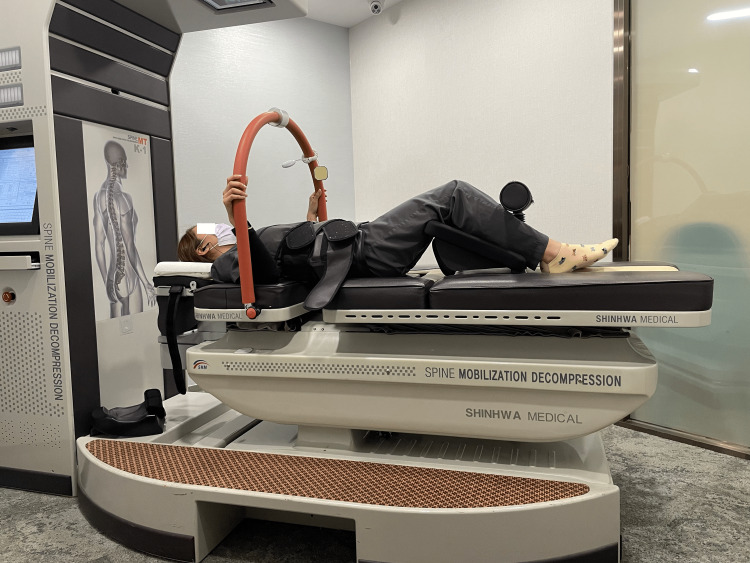
Non-surgical spinal decompression therapy (SpineMT; Shinhwa Medical, Busan, Korea). The patient is lying in supine position. Her upper torso is stabilized and knees are flexed in a knee rest. Pelvic belt was utilized to avoid rotations in the sacroiliac and pelvic joints. Upper torso belts are fitted in a comfortable position so the device gently tilted anteriorly to create flexion at L4/5 segment, and pulled on the patient's lower belt while the upper belt remains fixed, thereby decompressing L4/5 segments. The patient controls an emergency switch to immediately release all tension when needed.

The chiropractor prescribed a three-week treatment plan consisting of three visits per week. During each visit, the patient underwent spinal manipulation, non-surgical spinal decompression therapy (SpineMT; Shinhwa Medical, Busan, Korea) (Figure 2), and instrument-assisted soft tissue mobilization (IASTM) to correct spinal alignment and reduce disc pressure and muscle tenderness. The patient reported a successful reduction in his back pain, from 7/10 to 3/10 on the numeric scale, within the first three weeks of treatment. After the third week, the visit frequency was decreased to twice per week. Ergonomics advice, including proper standing posture, such as distributing the weight evenly between feet to avoid stooping forward, avoiding bending and heavy lifting, and taking frequent standing breaks. After three months, the patient's pain further decreased and his quality of life improved significantly; the World Health Organization Quality of Life score increased from 68% to 96%. Most importantly, the patient's range of motion had fully returned and he returned to work. The patient reported full recovery from his symptoms and physical functioning at work.

## Discussion

Diagnosing large LDH may be challenging, and severe disc herniation often mimics spinal tumors [6-10]. Thorough medical history, clinical features, physical examination, and radiological examinations such as an MRI or computed tomography are also used to diagnose and evaluate the condition. MRI is particularly helpful for evaluating primary or metastatic tumors, hematoma, and abscess in the differential diagnosis [8]. In our case study, MRI data revealed a radiological differential diagnosis that included both LDH and an epidural mass lesion. As a result, further MRI with contrast was performed to eliminate the possibility of a serious pathology. Although the prevalence of tumors for patients with low back pain presenting to chiropractic clinics is 0.03% [11], they have been constantly identified by chiropractors as primary care practitioners [12-14]. A greater understanding of the radiological and pathological characteristics of a severely herniated disc should reduce misdiagnoses and severe pathologies.

The fundamental challenge in managing patients with lumbar disc disease and neurological dysfunction is to correlate radiological findings with clinical history and symptoms to direct treatment and advice [15]. Radiological imaging can aid in differentiating between the two conditions, with disc herniation appearing as a focal protrusion of the disc material, whereas spinal tumors can appear as a mass lesion that can invade the surrounding bone or soft tissue. In our case study, the initial presentation seemed to be a spinal tumor, as the mass appeared to be large, and a follow-up scan with contrast ruled out the tumor option. It gives the clinician the diagnosis of a large L4/5 disc herniation, which is linked to mixed clinical symptoms; they also typically heal relatively quickly. In addition, the fragment exhibited a high T2 signal, indicating that it consisted primarily of water or nucleus pulposus material. As an updated study suggests that early resorption of LDH may occur in 24.7% of patients, the chiropractors anticipated that there is a high probability that the herniation may diminish naturally [16]. Although physical examinations used to identify LDH [17] are poorly correlated with diagnostic evidence, the use of MRI in the diagnosis of LDH has a high diagnostic value [18]. The efficacy of surgical and conservative treatments for symptomatic LDH is highly debatable. In a routine clinical setting, a recent study compared the long-term and short-term effectiveness of conservative and surgical treatments for quality of life and severity of sciatic symptoms in patients with LDH and found that there was no evidence to suggest that surgical treatment had any advantage over conservative treatment in long-term re-evaluations [19]. According to the majority of guidelines, if LDH is symptomatic after three months and conservative treatment is ineffective, surgical surgery may be recommended [16].

Chiropractic treatment included spinal manipulation, noninvasive spinal decompression therapy, and scraping therapy (IASTM). In randomized controlled studies for lumbar disc herniation, spinal manipulation was effective in relieving acute low back pain and sciatic symptoms [5,20], and non-surgical spinal decompression therapy had a positive impact on LDH in increasing disc height and resorption of herniation [21]. Correction of spinal alignment, reduction of muscular hypertonicity, breakup of myofascial adhesions, and release of nerve entrapments are the biomechanical outcome of chiropractic spinal manipulation on symptom reduction [22]. IASTM also reduces inflammatory cytokines, inhibits neuronal responses, creates antinociceptive effects of nitric oxide, and modulates pain by counterirritation to produce therapeutic advantages [23]. However, the cause of significant disc herniation and the method by which the symptoms are alleviated remain unknown in the present study without a control group. As this was a retrospective study, information regarding the diagnosis, treatment, and outcome monitoring was not always exhaustive.

## Conclusions

Large LDHs can be difficult to diagnose because they frequently mimic spinal tumors. MRI with contrast is particularly helpful when evaluating differential diagnoses. Severe disc herniation with a high T2 signal may heal quickly under conservative treatment, such as chiropractic therapy and lifestyle modifications. If conservative treatment fails, surgery may be necessary to remove the herniated disc and relieve pressure on the spinal nerve roots and spinal cord. Chiropractors should be able to use the diagnostic tools in making the proper management of LDH.

## References

[1] Kerr D, Zhao W, Lurie JD (2015). What are long-term predictors of outcomes for lumbar disc herniation? A randomized and observational study. Clin Orthop Relat Res.

[2] Wang W, Long F, Wu X, Li S, Lin J (2022). Clinical efficacy of mechanical traction as physical therapy for lumbar disc herniation: a meta-analysis. Comput Math Methods Med.

[3] Lee HW, Kwon YM (2013). Traumatic intradural lumbar disc herniation without bone injury. Korean J Spine.

[4] (2023). Herniated Disc. https://www.aans.org/Patients/Neurosurgical-Conditions-and-Treatments/Herniated-Disc#:~:text=A%20herniated%20disc%20is%20frequently,level%20of%20the%20disc%20herniation..

[5] Xu J, Ding X, Wu J (2020). A randomized controlled study for the treatment of middle-aged and old-aged lumbar disc herniation by Shis spine balance manipulation combined with bone and muscle guidance. Medicine (Baltimore).

[6] Li ST, Zhang T, Shi XW, Liu H, Yang CW, Zhen P, Li SK (2022). Lumbar disc sequestration mimicking a tumor: report of four cases and a literature review. World J Clin Cases.

[7] Li K, Li Z, Geng W, Wang C, Ma J (2016). Postdural disc herniation at L5/S1 level mimicking an extradural spinal tumor. Eur Spine J.

[8] Hoch B, Hermann G (2010). Migrated herniated disc mimicking a neoplasm. Skeletal Radiol.

[9] Song KJ, Kim KB, Lee KB (2012). Sequestrated thoracic disc herniation mimicking a tumoral lesion in the spinal canal--a case report. Clin Imaging.

[10] Lee JS, Suh KT (2006). Intradural disc herniation at L5-S1 mimicking an intradural extramedullary spinal tumor: a case report. J Korean Med Sci.

[11] Chu EC, Trager RJ (2022). Prevalence of serious pathology among adults with low back pain presenting for chiropractic care: a retrospective chart review of integrated clinics in Hong Kong. Med Sci Monit.

[12] Chu EC, Trager RJ, Yee WJ, Ng KK (2022). Lumbar schwannoma as a rare cause of radiculopathy in the chiropractic office: a case report. Cureus.

[13] Chu EC, Trager RJ, Lai CR, Leung BK (2022). Presumptive prostate cancer presenting as low back pain in the chiropractic office: two cases and literature review. Cureus.

[14] Chu EC, Trager RJ (2022). Thoracic schwannoma as an unusual cause of sciatic pain in the chiropractic office: a case report. Am J Case Rep.

[15] Li Y, Fredrickson V, Resnick DK (2015). How should we grade lumbar disc herniation and nerve root compression? A systematic review. Clin Orthop Relat Res.

[16] Hornung AL, Barajas JN, Rudisill SS (2023). Prediction of lumbar disc herniation resorption in symptomatic patients: a prospective, multi-imaging and clinical phenotype study. Spine J.

[17] van der Windt DA, Simons E, Riphagen II (2010). Physical examination for lumbar radiculopathy due to disc herniation in patients with low-back pain. Cochrane Database Syst Rev.

[18] Huang Z, Zhao P, Zhang C, Wu J, Liu R (2022). Value of imaging examinations in diagnosing lumbar disc herniation: a systematic review and meta-analysis. Front Surg.

[19] Gugliotta M, da Costa BR, Dabis E (2016). Surgical versus conservative treatment for lumbar disc herniation: a prospective cohort study. BMJ Open.

[20] Santilli V, Beghi E, Finucci S (2006). Chiropractic manipulation in the treatment of acute back pain and sciatica with disc protrusion: a randomized double-blind clinical trial of active and simulated spinal manipulations. Spine J.

[21] Choi E, Gil HY, Ju J, Han WK, Nahm FS, Lee PB (2022). Effect of nonsurgical spinal decompression on intensity of pain and herniated disc volume in subacute lumbar herniated disc. Int J Clin Pract.

[22] Onel D, Tuzlaci M, Sari H, Demir K (1989). Computed tomographic investigation of the effect of traction on lumbar disc herniations. Spine (Phila Pa 1976).

[23] Chu EC, Wong AY, Sim P, Krüger F (2021). Exploring scraping therapy: contemporary views on an ancient healing - a review. J Family Med Prim Care.

